# Asymmetric phenotype of Axenfeld-Rieger anomaly and aniridia associated with a novel *PITX2* mutation

**Published:** 2011-05-06

**Authors:** Simon K. Law, Maha Sami, Natik Piri, Anne L. Coleman, Joseph Caprioli

**Affiliations:** Jules Stein Eye Institute, David Geffen School of Medicine, University of California, Los Angeles, CA

## Abstract

**Purpose:**

To evaluate the asymmetry of the anterior segment phenotype between the two eyes of a patient with Axenfeld-Rieger syndrome (ARS).

**Methods:**

The entire database of a tertiary glaucoma practice was screened for patients with ARS. The medical records of patients with ARS were reviewed. The clinical characteristics of ocular examination of the two eyes of each patient were recorded and compared. Dental and medical information were also reviewed where available. The anterior segment phenotype was tabulated to assess asymmetry. Asymmetric anterior segment characteristics of patients with ARS were compared with reported cases in the literature.

**Results:**

Eight patients with ARS were identified from screening of more than 5,000 patients of a tertiary glaucoma practice. All patients had Axenfeld-Rieger anomaly in both eyes except one patient presented with an asymmetric phenotype of the anterior segment with features of Axenfeld-Rieger anomaly in one eye, but aniridia in the other eye. This patient had non-ocular findings including flat midface, hypodontia with lack of an upper incisor, and redundant periumbilical skin, typical for ARS. A heterozygous C>T nucleotide substitution was identified in exon 4 of the pituitary homeobox 2 (*PITX2*) gene, resulting in the replacement of a glutamine codon (CAG) with a stop codon (TAG) at amino acid position 67. This mutation is denoted c.199C>T at the cDNA level or p.Gln67Stop (or Q67X) at the protein level. Only three cases with asymmetric anterior segment phenotype between the two eyes of a patient with AGS have been reported in the literature.

**Conclusions:**

Variability in phenotype may occur between the two eyes of an individual affected by ARS. The current case undermines the advantage of genetic testing to correctly diagnose a rare disease.

## Introduction

Axenfeld-Rieger syndrome (ARS) is characterized by a variable combination of anterior segment dysgenesis, dental anomalies, and umbilical hernia. Reported anterior segment features in ARS include iris stromal hypoplasia (poor development of the anterior stroma of the iris), iridogoniodysgenesis (hypoplastic iris with abnormal “wooly” iridocorneal angle tissue), corectopia (eccentric pupil), polycoria (iris tears), ectropion uveae (eversion of the pupillary margin), posterior embryotoxon (prominent and centrally displaced Schwalbe’s line), and iris strands bridging the iridocorneal angle to the trabecular meshwork [1]. About half of the patients develop secondary angle closure glaucoma [2]. Although there are a few cases of an asymmetric phenotype between fellow eyes of a patient with ARS, similar anterior segment characteristics of fellow eyes are the usual presentation [3,4]. The purpose of this study was to evaluate the asymmetry of phenotype of the anterior segment between the two eyes of a patient with Axenfeld-Rieger syndrome (ARS), and present a case of ARS with asymmetric anterior segment phenotype and the associated genotype.

## Methods

The Institutional Review Board at University of California Los Angeles, Los Angeles, CA approved the current study and it was performed in accordance with the Declaration of Helsinki. The entire database of a tertiary glaucoma practice was screened to identify patients with ARS. The medical records of patients with ARS were retrospective reviewed. The medical data collected included the eye examination, and dental and medical records. The clinical characteristics of the two eyes of each patient with ARS were recorded and compared. The anterior segment phenotype was tabulated to assess asymmetry. A literature review of asymmetric phenotypes of the anterior segment of patients with ARS was conducted.

In review of the preliminary data collected, one patient had markedly asymmetric anterior segment phenotype between the two eyes. Genetic testing was ordered for this patient. PCR and DNA sequencing was performed by GeneDx (Gaithersburg, MD). Primer sequences and PCR conditions cannot be disclosed here since GeneDx has proprietary rights on this information.

## Results

Eight patients with ARS were identified from screening the medical records of more than five thousand patients of a tertiary glaucoma practice. All patients had symmetric Axenfeld anomaly and/or Rieger anomaly in both eyes, except for one patient who presented with an asymmetric phenotype of the anterior segment with Axenfeld-Rieger anomaly in one eye, but aniridia in the other eye. Table 1 summarizes the clinical characteristics of the ocular examination of the eight patients. Except for the case (Patient MA) with a markedly asymmetric phenotype between the two eyes, the rest of the patients had iris stromal hypoplasia, corectopia, polycoria, ectropion uveae, and posterior embryotoxon in both eyes. Seven of the eight patients had glaucoma in both eyes. One patient who had mild Axenfeld anomaly had no glaucoma in either eye. Six patients had central corneal thickness (CCT) measured for at least one eye. The average CCT (standard deviation) of the right eye was 602 (40) microns and left eye was 613 (57) microns.

**Table 1:** Clinical presentations of the two eyes of an individual with Axenfeld-Rieger syndrome.

**Patient**	**Year of Birth**	**Sex**	**Anterior segment phenotype of first eye**	**Anterior segment phenotype of second eye**	**Additional ocular findings**	**Eye with Glaucoma**	**Central corneal thickness (Right/Left eye) in micron**	**Dental Abnormalities**	**Umbilical Abnormalities**
G.G.	1980	Male	Axenfeld & Rieger anomalies	Axenfeld & Rieger anomalies		Both eyes	560 / 545	Unknown	Unknown
K.M.	1980	Male	Rieger anomaly	Rieger anomaly	Optic nerve coloboma (right eye)	Both eyes	616 / 616	Unknown	Unknown
J.B.*	1990	Female	Axenfeld anomaly	Axenfeld anomaly	Microcornea and scleral thinning (both eyes)	None	576 / 606	Presence	Presence
M.B.*	1960	Female	Axenfeld & Rieger anomalies	Axenfeld & Rieger anomalies	Corneal scar and scleral thinning (left eye)	Both eyes	571 / left corneal scar	Presence	Unknown
A.C.	1954	Male	Axenfeld & Rieger anomalies	Axenfeld & Rieger anomalies		Both eyes		Unknown	Unknown
A.M.	2009	Female	Rieger anomaly	Rieger anomaly	Cataract and corneal scar (both eyes)	Both eyes		Unknown	Absence
H.L.	1996	Male	Axenfeld & Rieger anomalies	Axenfeld & Rieger anomalies		Both eyes	626 / not measurable	Presence	Presence
M.A.	2003	Female	Axenfeld & Rieger anomalies	Aniridia		Both eyes	664 / 683	Presence	Presence

Signs of Axenfeld anomaly include: prominent schwalbe line, iris adhesion to cornea and trabecular meshwork. Signs of Rieger anomaly include: iris hypoplasia, correctopia or polycoria, ectropion uveae. *J.B. and M.B. had a mother-and-son relationship

In this retrospective review, the seven patients that had bilateral ocular findings typical for ARS received no genetic work up. However, the patient with the unusual asymmetric phenotype with features of Axenfeld-Rieger anomaly in one eye and aniridia in the other eye posed a diagnostic uncertainty and systemic and genetic evaluation were performed. Details of the clinical presentation and evaluation of this patient are reported as follows.

### Case (Patient M.A.)

**Figure 1:**
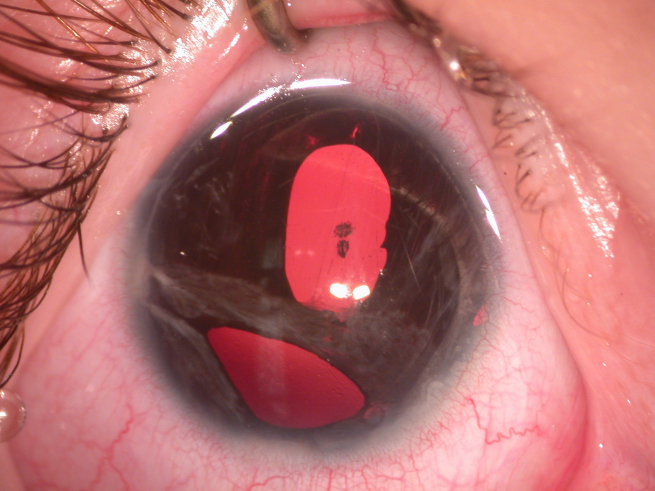
Anterior segment photograph of the right eye shows polycoria, adhesion of iris strands with peripheral cornea, and posterior emblyotoxon.

**Figure 2:**
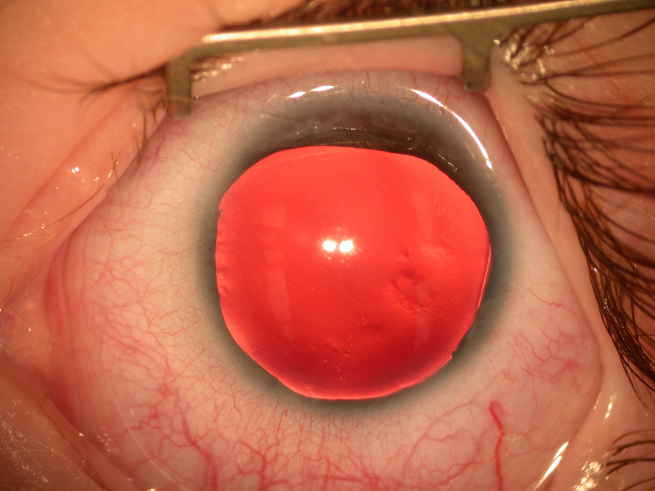
Anterior segment photograph of the left eye shows aniridia.

**Figure 3:**
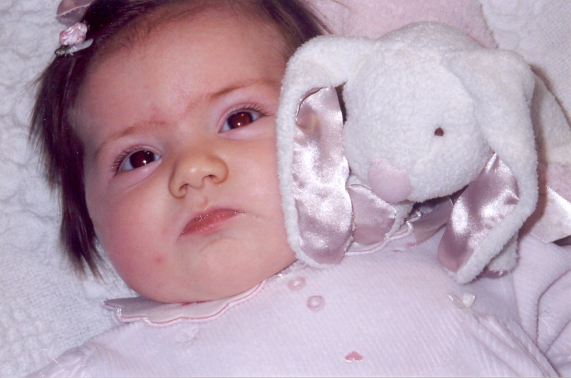
Old photograph of patient when she was 3 months old shows similar anterior segment appearance.

**Figure 4:**
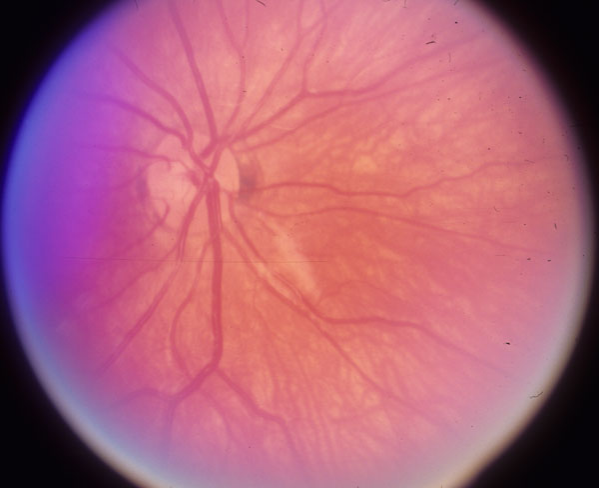
Optic disc photograph of the right eye shows normal neural rim.

**Figure 5:**
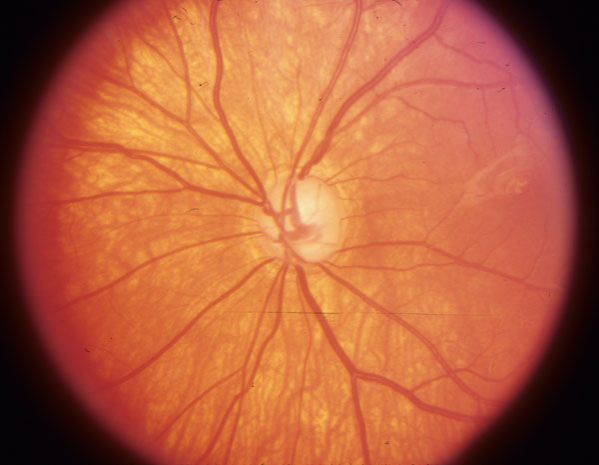
Optic disc photograph of the left eye shows severe loss of neural rim.

**Figure 6:**
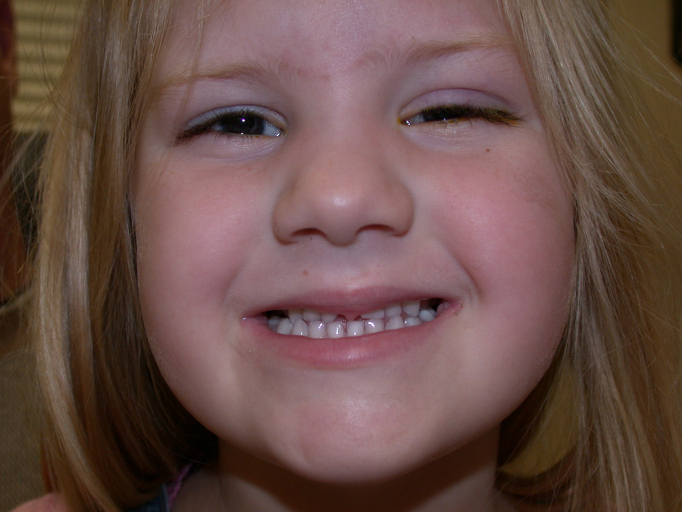
Facial photograph of patient shows flat midface and hypodontia lacking of an upper incisor.

**Figure 7:**
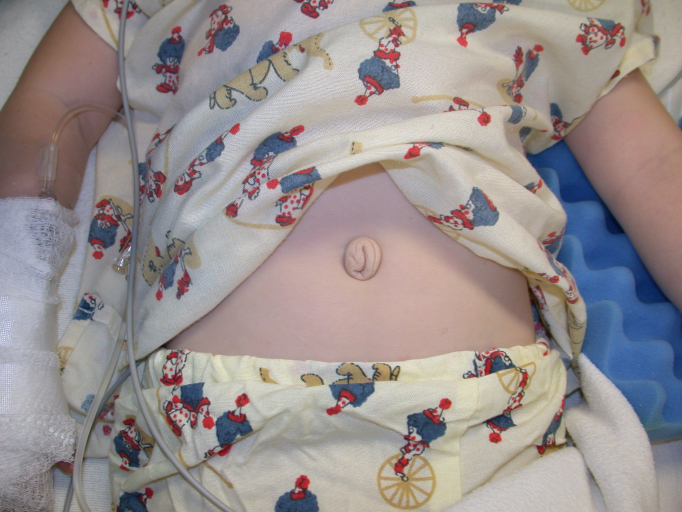
Abdominal photograph of patient shows redundant periumbilical skin.

The patient was referred to our institute when she was four years old with an uncertain diagnosis of ARS versus aniridia. Shortly before the referral, she was found to have an increased intraocular pressure (IOP) in both eyes: 27 mmHg in the right and 46 mmHg in the left, and was started on glaucoma medications. When she was using timolol and dorzolamide as fixed combination and bimatoprost eye drops in both eyes, the IOP measured by Goldmann tonometry was 14 mmHg in the right and 32 in the left. Her spectacle-corrected vision was 20/80 in the right and 20/40 in the left, with moderate hyperopia in the right and mild myopia in the left. The right eye was amblyopic, and was undergoing occlusion therapy. She has no strabismus or nystagmus. Slit lamp examination of the right eye revealed polycoria, adhesion of iris strands to the peripheral cornea, and posterior embryotoxon. (Figure 1) Examination of the left eye revealed a small iris stub resembling aniridia, and a similar appearance was noted on an old photograph taken when she was 3 months old (Figure 2 and Figure 3).Corneal pachymetry was 664 microns in the right and 683 microns in the left. The horizontal corneal diameter measured 11 mm in both eyes. Axial length by ultrasonic measurement was 21.78 mm in the right eye and 24.46 mm in the left eye. Gonioscopy revealed extensive iris strands bridging the iridocorneal angle to the trabecular meshwork in the right and complete angle closure by the stub of the iris in the left. Optic disc examination revealed a cup/disc ratio of 0.30 in the right eye and 0.95 in the left, and there was no retinal pathology (Figure 4 and Figure 5). Further physical examination revealed a flat midface, hypodontia with lack of an upper incisor, and redundant periumbilical skin (Figure 6 and Figure 7). Ultrasonic examination of kidneys was normal and there was no cardiac abnormality. She has normal intelligence. Other family members including both parents and a younger brother were examined and no similar anomaly was noted. However, the maternal uncle has Russell-Silver syndrome and was not available to be examined.

**Figure 8:**
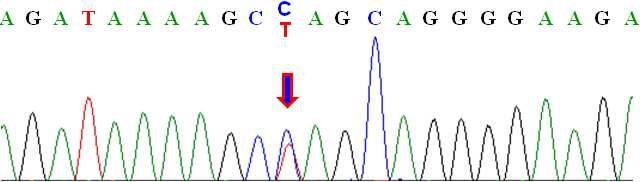
Sequencing analysis of the Axenfeld-Rieger syndrome patient’s *PITX2* gene with the C>T mutation.

Parents of the patient underwent genetic counseling and consented to the genetic testing of the patient. Genomic DNA from the patient’s blood sample was PCR-amplified and screened for mutations in pituitary homeobox 2 (*PITX2*), forkhead box C1 (*FOXC1*), and paired box 6 (*PAX6*) genes that are known to be associated with ARS. Coding exons of *PITX2b* (NM_153426) were analyzed in this study. A heterozygous C>T nucleotide substitution (a nonsense mutation) was identified in exon 4 of *PITX2*, resulting in the replacement of a glutamine codon (CAG) with a stop codon (TAG) at amino acid position 67. This mutation is denoted c.199C>T at the cDNA level or p.Gln67Stop (or Q67X) at the protein level (Figure 8). No sequence alterations were detected in *FOXC1* and *PAX6*.

She subsequently received a tube shunt procedure in the left eye and an additional tube shunt procedure 2 years later with pressure of the left eye controlled at 20–22 mmHg on minimum topical medication.

### Literature review

A literature review identified asymmetric phenotypes of the anterior segment in three patients with ARS (Table 2). In 1883, Vossius [5] described a nine-year-old girl with bilateral iris defects associated with dental anomalies. One eye had an ectopic pupil (corectopia) with full-thickness iris stromal defects (polycoria) while the fellow eye had a near-total absence of iris tissue (aniridia). The other two patients were reported by Perveen et al. [4] in 2000. These two patients had unilateral Peters anomaly in one eye but the other eye had Rieger anomaly or iris hypoplasia [4].

**Table 2:** Clinical presentations and mutations of cases with asymmetric anterior segment phenotype between the two eyes of an individual with Axenfeld-Rieger syndrome [1,4,5].

**Reference**	**Anterior segment phenotype of first eye**	**Anterior segment phenotype of second eye**	**Additional ocular findings**	**Glaucoma**	**Dental abnormalities**	**Umbilical abnormalities**	**Mutation**
[5]	Near total absence of iris (aniridia)	full thickness iris stromal defects	Unknown	Unknown	Unknown	Unknown	Unknown
[4]	Peters anomaly	Rieger anomaly, anterior polar cataract	Mild unilateral foveal hypoplasia	Absence	Presence	Presence	PITX2 Ivs 3 (−2); A>T
[4]	Axenfeld anomaly, iris hypoplasia	Peters anomaly	None	Absence	Presence	Unknown	PITX2 C ins 1083
Current patient	Rieger anomaly, Axenfeld anomaly	Aniridia	None	Presence	Presence	Presence	PITX2 c. 199 C>T (Q67X)

Signs of Axenfeld anomaly include: prominent schwalbe line, iris adhesion to cornea and trabecular meshwork. Signs of Rieger anomaly include: iris hypoplasia, correctopia or polycoria, ectropion uveae. Signs of Peters anomaly were not explained in the report by Perveen et al. [4].

## Discussion

Although tremendous variability in phenotype within a single family with ARS has been observed, phenotypes between the two eyes of an affected individual are usually similar [1]. Seven of the eight patients with ARS in our tertiary glaucoma practice presented with a similar anterior segment phenotype between the two eyes. However, one patient presented with anterior segment features of Axenfeld-Rieger anomaly in one eye, and aniridia in the other eye. In 1883, Vossius [5] described a nine-year-old girl with Rieger anomaly in one eye including an ectopic pupil with full-thickness iris stromal defects, while the fellow eye had a near-total absence of iris tissue (aniridia). To our knowledge, description of the asymmetric phenotype of Axenfeld-Rieger anomaly in one eye and severe iris hypoplasia resembling aniridia in the other eye has not been previously reported.

It is known that mutations in the transcription factor *PITX2* and *FOXC1* genes lead to ARS, while mutations of the *PAX6* gene underlies many cases of aniridia [6,7]. However, Henkind and associates [8] described a family with ARS that included two members who appeared to have aniridia. In addition, cases of ARS have been described associated with a deletion of *PAX6* [6,9,10], and cases of bilateral aniridia with proven *PITX2* and *FOXC1* mutations [4,11]. Because of the mixed anterior segment anomalies between the two eyes of this patient and the association of Wilm’s tumor in sporadic cases of aniridia, genetic testing was conducted for *PITX2*, *FOXC1*, and *PAX6*  [6]. The nonsense mutation, Q67X, in exon 4 of *PITX2* identified has not previously been reported, to our knowledge [7].

*PITX2* mutations are primarily responsible for ARS but have also been associated with Peters anomaly, iris hypoplasia/iridogoniodysgenesis syndrome, aniridia, and ring dermoid of the cornea [4,7,12]. Intragenic mutations of *PITX2* have been described in more than 40 ARS patients to date and include missense, nonsense, splice-site mutations, and deletions/insertions/duplications. There is no apparent correlation between the mutation location in *PITX2* and the severity of the clinical phenotype. However, the characterization of several missense mutations indicates that the severity of the phenotype can be correlated to the remaining function of the mutant PITX2. In heterozygotes, the function of one wild-type PITX2 allele alone results in the severe ARS form. Contribution of the mutant allele would result in the milder iris hypolasia or iridogoniodysgenesis syndrome forms, depending on the degree of residual activity [13]. A nonsense mutation in *PITX2* found in patient M.A. is predicted to result in degradation of the mutant mRNA through the nonsense-mediated decay pathway that prevent the synthesis of the truncated protein [14]. Even if the truncated 66 amino acid (aa) long protein is synthesized and stable, it would be shorter than the native protein by 251 aa and would lack helix 2 and helix 3/4 of the DNA binding homeodomain involved in transcriptional regulation and the OAR domain in the COOH-terminal end that has been proposed to increase the DNA binding and transcriptional activity of PITX2 during development [15]. The absence of clinical and genetic abnormalities in other family members suggests that this may be a de novo genomic alteration [3]. Finally, since PITX2 is known to inhibit FOXC1 activity [16], the mechanism for PITX2-related severity of the ocular phenotype in this patient may be the simultaneous consequence of PITX2 haploinsufficiency and a gain of function of FOXC1.

Mutations of *PITX2* are usually associated with full-spectrum ARS phenotypes that include both ocular and nonocular features, as in patient M.A [1]. Intriguingly, PITX2 is also involved in left-right polarity determination, yet asymmetry defects are not a usual feature of ARS [3,4,17]. Iris abnormalities are usually stationary, but rarely have been demonstrated to change over time [18]. In our patient, the aniridia in the left eye appears to remain unchanged since infancy by comparison with old photographs. Aniridia is a panocular disease with associated ocular and visual defects such as corneal opacification, cataract, foveal dysplasia, optic nerve hypoplasia, and nystagmus, but none of which is found in this patient [6]. It is possible that the different phenotypes of iris abnormality in the two eyes of this patient fall within a spectrum of iris defect in ARS.

The major clinical concern in ARS is the risk of developing sight-threatening glaucoma, which is estimated to occur in 50% of the patients [1,2]. Although physical occlusion of the angle structure is not prerequisite for elevated IOP and glaucoma in ARS, the severity of glaucoma does correlate with the level of iris insertion into the angle [2]. In the seven patients of this series who had glaucoma in both eyes, the eye with more extensive areas of angle closure presented with higher IOP and was more resistant to medical and surgical management.

Patients in this series had a thicker central cornea compared to normal adults, and a thick central cornea is associated with artificially higher IOP estimation by applanation. Pachymetry of patients with ARS is rarely reported in the literature. In mouse models with overexpressing PITX2A isoform in the cornea, corneal hypertrophy was noted together with iridocorneal adhesion, gray and tearing eyes, and severe apoptosis-associated retinal degeneration [19]. In human with ARS-causing PITX2 mutation, V45L, elevated transactivating properties in cell culture were found [20]. It is suggestive that increased as well as reduced PITX2 activities are deleterious during development [17]. Further functional studies may clarify if the novel mutation of PITX2 found in our patient is associated with a gain-of-function leading to an increase of thickness of the cornea.

In summary, although phenotypic presentation of ARS is highly variable within a single family, only a few cases in the literature had different phenotypes between the two eyes of an individual. We presented a case of an asymmetric phenotype of Axenfeld-Rieger anomaly in one eye and aniridia in another of a patient with a novel nonsense point mutation of *PITX2*. The current case undermines the variability in phenotype even between the two eyes of an individual affected by ARS and the advantage of genetic testing in correctly diagnosing a rare disease.
